# Home delivery of medication during Coronavirus disease 2019, Cape Town, South Africa: Short report

**DOI:** 10.4102/phcfm.v12i1.2449

**Published:** 2020-06-04

**Authors:** Zameer Brey, Robert Mash, Charlyn Goliath, Darrin Roman

**Affiliations:** 1 Bill and Melinda Gates Foundation, Cape Town, South Africa; 2 Division of Family Medicine and Primary Care, Faculty of Medicine and Health Sciences, Stellenbosch University, Cape Town, South Africa; 3 Metropolitan Health Services, Western Cape Government, Cape Town, South Africa

**Keywords:** primary care, COVID-19, community health workers, chronic disease, medication adherence

## Abstract

The public sector primary care facilities in Cape Town serve a large number of patients with chronic diseases such as human immunodeficiency virus, tuberculosis, diabetes, hypertension, asthma and chronic obstructive pulmonary disease. Prior to the Coronavirus disease 2019 (COVID-19) epidemic, stable patients with chronic conditions attended the facility or support groups to obtain their medication. During the COVID-19 epidemic, these patients would be put at risk if they had to travel and gather in groups to receive medication. The Metropolitan Health Services, therefore, decided to offer home delivery of medication. A system of home delivery was rapidly established by linking the existing chronic dispensing unit system with the emerging approach to community-orientated primary care in the Metro. Medication was delivered as usual to primary care pharmacies, but then a variety of means were used to disseminate the parcels to local non-profit organisations, where they could be delivered by a city-wide network of community health workers (CHWs). Innovations included various ways of delivering the parcels, including via Uber, bicycles and electric scooters, as well as Google forms to monitor the success of the initiative. It was estimated that up to 200 000 parcels per month could be delivered in this way via 2500 CHWs. The new system was established throughout the Metropole, and its strengths, weaknesses, opportunities and threats are further discussed. The initiative may prevent COVID-19 amongst people with comorbidities who would be at risk of more severe diseases. It may also have de-congested primary care facilities ahead of the expected surge in COVID-19 cases.

## Introduction

Public sector primary care facilities in Cape Town care for a large number of people with chronic conditions such as human immunodeficiency virus (HIV), tuberculosis, diabetes, hypertension, asthma and chronic obstructive pulmonary disease.^1^ Many of these patients are at an increased risk of more severe Coronavirus disease 2019 (COVID-19) infection.^2^ The public sector also serves the lower socio-economic sections of society, those without health insurance, who may also be more vulnerable because of poverty, overcrowding in informal settlements or malnutrition. In addition, the average age of patients with chronic non-communicable diseases is over 50 years, which also puts this group at risk of more severe COVID-19 infection.^1^ This group of patients with chronic conditions is therefore a particularly vulnerable group during the epidemic.

Under normal circumstances, the Metropolitan Health Services (MHS) estimate that 200 000 of these more stable and better-controlled patients receive pre-packaged medication from the facility’s pharmacy or via support groups and clubs each month. In collecting their medication, they are exposing themselves to larger groups of people when they commute using congested public transport, wait in queues at facilities and/or participate in group activities, enabling rapid spread of the virus.

## Home delivery

**FIGURE 1:**
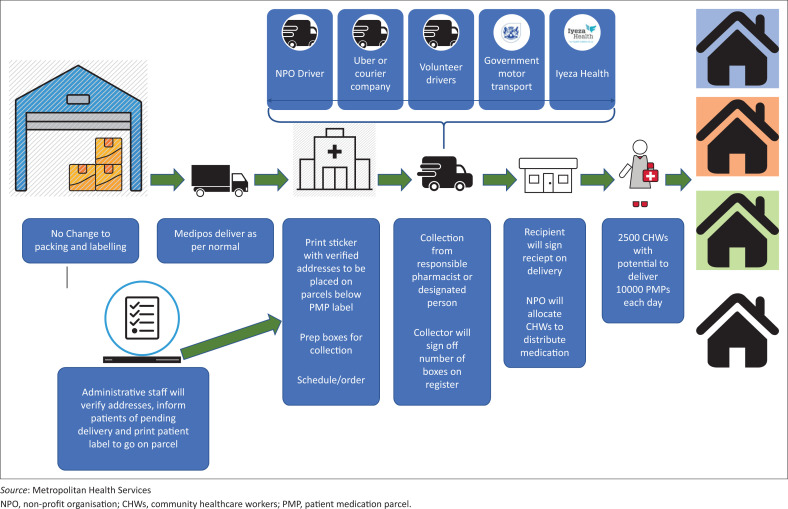
System for delivering medication at home.

The MHS decided to institute home delivery of medication to reduce the risk of COVID-19 in this vulnerable group of patients and thereby reduce the overall risk of transmission associated with the movement of people. The system consisted of the following steps (Figure 1):

1. A central dispensing unit (CDU) pre-packaged medication.
2. A private company delivered these to pharmacies at primary care facilities.
3. A team at each primary care facility called patients to verify their addresses and printed address labels to attach to the medication parcels.
4. Pharmacists and their teams organised the parcels into boxes by geographic area.
5. Drivers delivered the boxes to non-profit organisations (NPOs).
6. Non-profit organisations allocated the medication to community health workers (CHWs) according to the area they typically worked in.
7. Community health workers delivered medication to the household and returned any undelivered medication.
8. The NPOs returned undelivered medication to the pharmacy at the primary care facility.

The system was conceptualised by the authors for the MHS and coordinated by a team of administrative and support staff from the substructures. Once established, the co-ordination was delegated to the substructures.

The system (Figure 1) required a number of innovations to make it functional. A key challenge was delivering medication from the pharmacy to the NPO. In many cases, the department used Uber drivers to collect and deliver medication. This required establishing and training teams of people in each substructure to arrange Uber drivers using a centralised Uber information system. This was sponsored by Uber SA during the lockdown period. At other facilities, the NPO came to collect medication or the facility had a driver who could deliver. In some areas, volunteers or organisations stepped in to assist with transport. In two communities, a local entrepreneur had established a small business (Iyeza Health) to deliver medication at home via bicycles or electric scooters for a small fee.

The system had virtually no incremental costs as the NPOs, CHWs and CDU were already financed. The only incremental costs were for transport from the pharmacy to the NPO. These costs were minimal as, for example, the cost of using Uber per parcel was $0.04.

Monitoring and evaluation of the system also required daily data on parcels received at the pharmacy, sent to NPOs and either delivered or returned. A simple Google form was created for pharmacists to report their data and collate this centrally.

## Strengths and weaknesses

The system relied on a number of subsystems that were already established. The CDU and delivery of pre-packaged medication to pharmacies had been operational for several years.

The MHS had also put energy into reorganising the primary healthcare services according to the principles of community-orientated primary care over the previous 2 years.^3^ This meant that there was an established network of NPOs and 2500 CHWs across the city with contiguous boundaries and delineated household areas. Each CHW was responsible for approximately 250 households, and they were grouped into teams of 10–15 CHWs each with a professional nurse coordinator. Changing the scope of practice of CHWs to deliver medication was therefore a feasible shift in task. The only missing link in the whole system was the delivery of medication from the pharmacy to the NPO.

It was estimated that 200 000 parcels needed to be delivered per month, which extrapolated to four parcels per CHW per day. This was a feasible goal and still left room for other essential tasks by the CHWs. After the first week of implementation, over 3000 parcels were being delivered per day.

The teams of people phoning patients discovered that few patients answered their phones and many addresses were incorrect. This manual process was problematic and existing patient records were incomplete. A public call by the Department of Health for people to verify their addresses was made and supported by a WhatsApp Clinic BOT and Unstructured Supplementary Service Data (USSD)-based automated questionnaire. A few patients had moved to other addresses during the lockdown, and parcels needed to be redirected, which was not easily accommodated by the system.

Another issue amongst HIV-positive patients was that home delivery could lead to inadvertent disclosure of HIV status to family members. Clerks from the HIV adherence clubs were tasked with verifying addresses and obtaining consent from patients for home delivery.

## Opportunities and threats

The major benefits of home delivery were reducing the spread of COVID-19 amongst a susceptible population. The initiative also has significant benefits for patients in the longer term as home delivery can avoid time away from work and loss of earnings to collect medication. Patient responses thus far have been encouraging.

The engagement of CHWs, professional nurses and NPOs in this important activity should also lead to improved relationships between facility-based and community-based healthcare workers, which was a weakness in the implementation of community orientated primary care (COPC). In addition, the stature and acceptability of CHWs within communities could be enhanced by performing such a valuable task.

Home delivery of medication also allowed CHWs to opportunistically screen households for symptoms of COVID-19 and to identify other health and social risks.

The same system can be used to deliver non-pharmaceutical materials, such as stoma and incontinence devices, or nutritional supplements.

Threats include a significant perceived change in the scope of practice of CHWs that might be contrary to the principles of COPC^3^ if they spend too much time delivering medication and performing outreach for the facility-based primary care services. This might detract from the more important focus on broader primary health care, community diagnosis, routine household assessment, health promotion and disease prevention.

Another threat to the success of the system is the complexity of relationships and the need for good communication and coordination between role-players and managers. The system requires coordination between CDU, facility managers, pharmacists, delivery drivers, NPO professional nurses and CHWs. It also requires communication and support from a variety of managers responsible for pharmacy, primary care, community-based services and NPOs.
